# Supplementary material for: The non-redundant functions of PIWI family proteins in gametogenesis in golden hamsters
Source: Nat Commun. 2023 Aug 29;14:5267. doi: 10.1038/s41467-023-40650-x (PMC10465502; doi:10.1038/s41467-023-40650-x)
Supplement: Supplementary file 1 — Supplementary Information [file 41467_2023_40650_MOESM1_ESM.docx]

**The non-redundant functions of PIWI family proteins in gametogenesis in golden hamsters**

Xiaolong Lv^1,#^, Wen Xiao^1,#^, Yana Lai^2,#^, Zhaozhen Zhang^2^^,#^, Hongdao Zhang^1,#^, Chen Qiu^2^, Li Hou^1^, Qin Chen^2^, Duanduan Wang^1^, Yun Gao^2^ ,Yuanyuan Song^1^, Xinjia Shui^1^, Qinghua Chen^2^, Ruixin Qin^2^, Shuang Liang^2^, Wentao Zeng^2^, Aimin Shi^2,*^, Jianmin Li^2,*^，Ligang Wu^1,*^

1. State Key Laboratory of Molecular Biology, Shanghai Key Laboratory of Molecular Andrology, Center for Excellence in Molecular Cell Science, Shanghai Institute of Biochemistry and Cell Biology, Chinese Academy of Sciences, University of Chinese Academy of Sciences, Shanghai 200031, China
2. State Key Laboratory of Reproductive Medicine and Offspring Health, Jiangsu Laboratory Animal Center, Jiangsu Animal Experimental Center of Medicine and Pharmacy, Department of Cell Biology, Animal Core facility, Key Laboratory of Model Animal, Collaborative Innovation Center for Cardiovascular Disease Translational Medicine, Nanjing Medical University, Nanjing 211166, China

# These authors contributed equally to this work.

* Correspondence:

Ligang Wu, [lgwu@sibcb.ac.cn](mailto:lgwu@sibcb.ac.cn)

Jianmin Li, [jianminlilab@njmu.edu.cn](mailto:jianminlilab@njmu.edu.cn)

Aimin Shi, sam@njmu.edu.cn


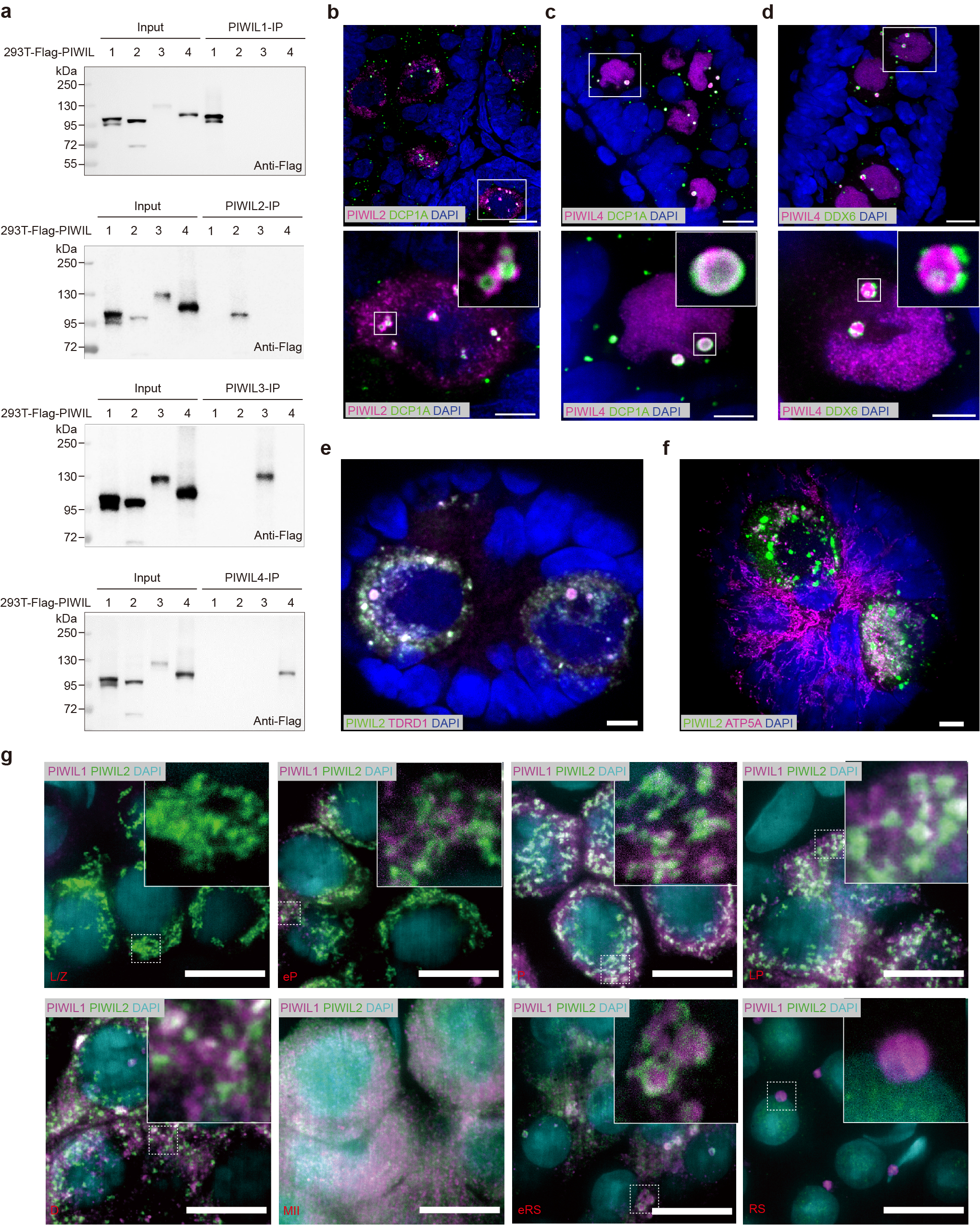


**Supplementary Fig. 1 Expression and location of PIWIs in male germ cells**

**(a)** Verification of antibody specificity for detection of PIWL1, PIWIL2, PIWIL3, and PIWIL4 in golden hamsters. Flag-tagged PIWIL1, PIWIL2, PIWIL3, and PIWIL4 were overexpressed in 293T cells, then immunoprecipitated using anti-PIWIL1, anti-PIWIL2, anti-PIWIL3, or anti-PIWIL4 antibodies, respectively. Detection of the immunoprecipitated products using anti-Flag antibody confirmed the specificity of anti-PIWI antibodies for their respective targets.

**(b-d)** Immunofluorescence staining of postnatal testes with anti-PIWIL2 and anti-DCP1A **(b)**, anti-PIWIL4 and anti-DCP1A **(c)**, or anti-PIWIL4 and anti-DDX6 **(d)** antibodies, respectively. Scale bar = 10 µm (top); Scale bar = 4 µm (bottom).

**(e-f)** Immunofluorescence staining of postnatal testes with anti-PIWIL2 and anti-TDRD1 **(e)** or anti-ATP5A **(f)** antibodies. Scale bar = 4 µm.

**(g)** Immunofluorescence staining of spermatocytes and spermatids with anti-PIWIL1 and anti-PIWIL2 antibodies. L/Z, leptotene/zygotene; eP, early pachytene; P, pachytene; LP, late pachytene; D, diplotene; MI/MII, meiosis I/meiosis II; eRS, early round sperm; RS, round sperm. Scale bar, 8 µm.

Source data are provided as a Source Data file.


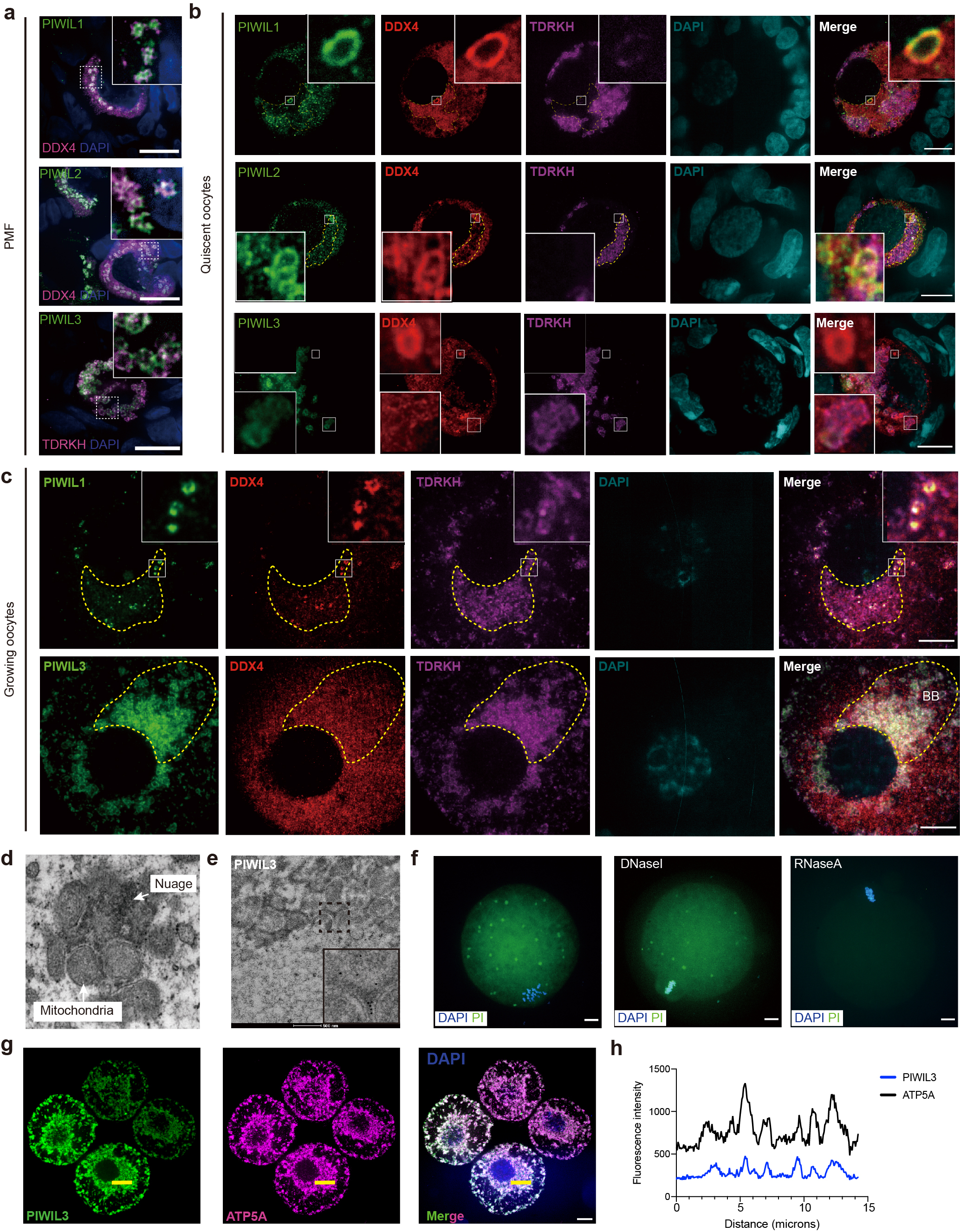


**Supplementary Fig. 2 Expression and location of PIWIs in female germ cells**

**(a)** Immunofluorescence staining showing the expression and location of PIWIL1, PIWIL2, or PIWIL3 in primordial follicles (PMF). PIWIL1 and PIWIL2 localized to inter mitochondrial cement (IMC), while PIWIL3 presents outer of mitochondria (OM) and co-localized with TDRKH. These two localizations are consistent with the contour of mitochondria aggerates. Scale bar, 8 µm.

**(b)** Immunofluorescence staining showing the location of PIWIL1, PIWIL2, and PIWIL3 in quiescent oocytes. Prominent perinuclear circle granules encompassed DDX4, PIWIL1, and PIWIL2, other than PIWIL3 and TDRKH. Scale bar, 4 µm.

**(c)** Immunofluorescence staining showing the location of PIWIL1 and PIWIL3 in growing oocytes. PIWIL1 colocalizes with DDX4 and TDRKH and forms prominent granules, while PIWIL3 colocalizes with TDRKH and presents a structure that resembles the Balbiani body (BB). Scale bar, 8 µm.

**(d)** Transmission electron micrograph showing the mitochondria and nuage. Scale bar, 0.5 µm.

**(e)** Immuno-gold labeling showing PIWIL3 presents in IMC in quiescent oocytes. Scale bar, 0.5 µm.

**(f)** DAPI and Propidium Iodide (PI) staining of MII oocytes treated with or without DNase I or RNase A show that dozens of prominent granules in the cytoplasm of MII oocytes are RNA granules. Scale bar, 10 µm.

**(g)** Immunofluorescence staining of four-cell embryos with anti-PIWIL3 and anti-ATP5A antibodies to determine their colocalization. Scale bar, 4 µm.

**(h)** Fluorescence intensity profiles of PIWIL3 and ATP5A within the yellow region of **(g)**. Overlapping peaks indicate colocalization.


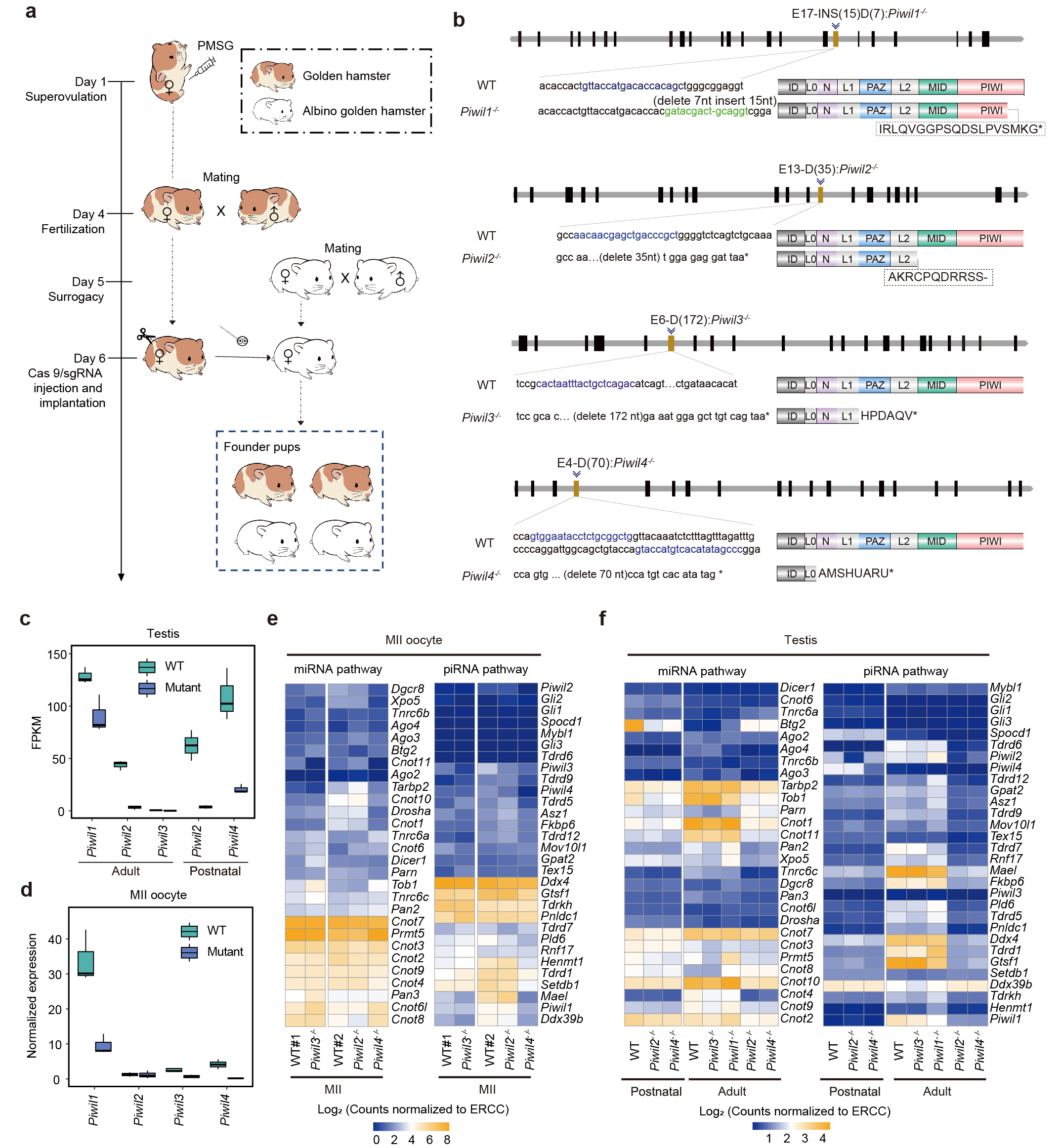


**Supplementary Fig. 3 Generation and validation of *Piwi*-deficient golden hamsters**

**(a)** Strategy to generate four *Piwi* gene mutant golden hamsters by two-cell embryo CRISPR-Cas9 injection. Cas9 mRNAs and sgRNAs were injected into two-cell embryos of WT golden hamsters, which were then transplanted to albino surrogate females mated with albino male hamsters to obtain *Piwi*-deficient founder pups.

**(b)** Structures of the golden hamster *Piwil1*, *Piwil2*, *Piwil3*, and *Piwil4* genes and target locus for sgRNAs. Black boxes denote the exons, and yellow boxes denote the target regions. *Piwil1^-/-^* strains contained a 7-nt deletion and a 15-nt insertion in exon 17 as reported before. *Piwil2^-/-^* strains contained a 35-nt deletion in exon 13. *Piwil3^-/-^* strains contained a 172-nt deletion in exon 6. *Piwil4^-/-^* strains contained a 70-nt deletion in exon 4. All mutations resulted in a frameshift in the relative *Piwi* mRNAs resulting in a premature stop codon.

**(c-d)** Validation of disrupted *Piwi* gene expression in *Piwi*-deficient testes **(c)** and MII oocytes **(d)** using RNA sequencing. Gene expression levels in testes are normalized to total mapped reads; gene expression levels in MII oocytes are normalized by ERCC spike-in. In box plots, the centre line represents the median value, the box borders represent the upper and lower quartiles (25th and 75th percentiles, respectively), and the ends of the top and bottom whiskers represent maximum and minimum scores, respectively. Data are means of the biological replicates for each mutant.

**(e-f)** Heatmaps of miRNA- and piRNA-related gene expression levels in WT and *Piwi*-deficient MII oocytes **(e)** and testes **(f)**. Gene expression levels are normalized by ERCC spike-in.

Data are the average values of the biological replicates for each mutant: n = 4 (WT#1 MII oocyte), n = 3 (WT#2 MII oocyte), n = 3 (*Piwil1*^-/-^ MII oocyte), n = 3 (*Piwil2*^-/-^ MII oocyte), n = 4 (*Piwil3*^-/-^ MII oocyte) or n = 2 (*Piwil4*^-/-^ MII oocyte); n = 2 (WT postnatal testes), n = 2 (*Piwil2*^-/-^ postnatal testes), n = 2 (*Piwil4*^-/-^ postnatal testes); n = 2 (WT adult testes), n = 2 (*Piwil1*^-/-^ adult testes), n = 2 (*Piwil2*^-/-^ adult testes), n = 2 (*Piwil3*^-/-^ adult testes).


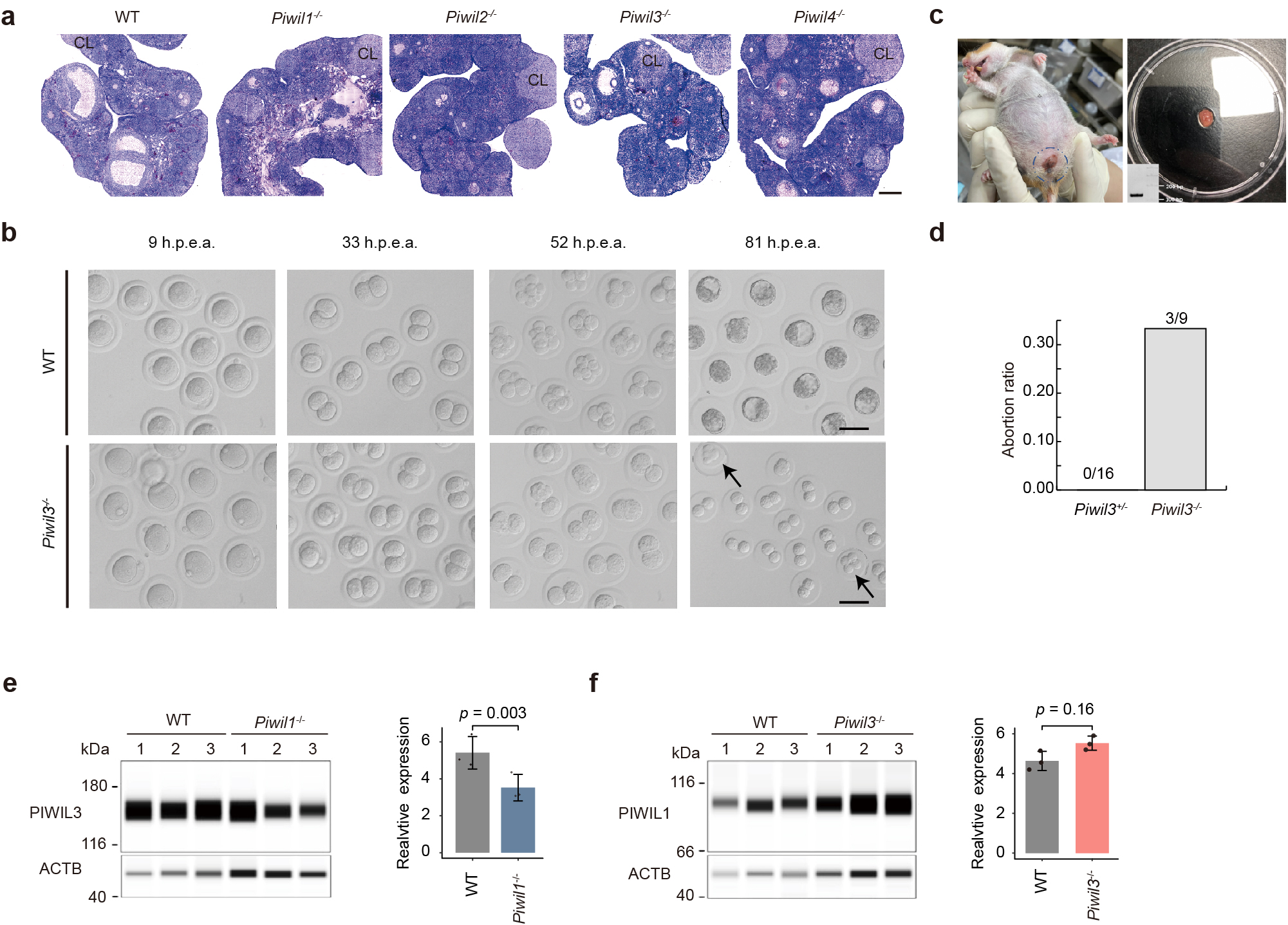


**Supplementary Fig. 4** **Female fertility phenotypes of *Piwi*-deficient golden hamsters**

**(a)** PAS staining of WT and *Piwi*-deficient ovaries. CL, corpus luteum. Scale bar, 100 µm.

**(b)** Representative images of *in vitro* cultured embryos at 9, 33, 52, and 81 h.p.e.a obtained from WT and *Piwil*3*^-/-^* oocytes fertilized *in vivo* with wild-type sperm. Scale bar, 100 µm.

**(c)** Representative images showing the aborted embryos with vaginal bleeding (left) and embryo fragments (right).

**(d)** Histogram showing the ratio of golden hamsters with aborted embryos.

**(e-f)** PIWIL3 protein levels in WT and *Piwil1*^-/-^ MII oocytes **(e)** and PIWIL1 levels in WT and *Piwil3*^-/-^ mutant MII oocytes **(f)**. 17-20 oocytes from 4–6-month-old hamsters were collected and lysed for protein analysis. Three biological replicates of oocytes were collected from each of three WT, *Piwil1*^-/-^, or *Piwil3*^-/-^ hamsters (n=2.7-3.3 oocytes/genotype). Signal intensity (area) shows PIWIL1 or PIWIL3 protein accumulation detected by capillary electrophoresis Western Blot assays. Significance was determined by unpaired two-tailed *t*-test. Data represent mean ± s.e.m (n = 3).

Source data are provided as a Source Data file.


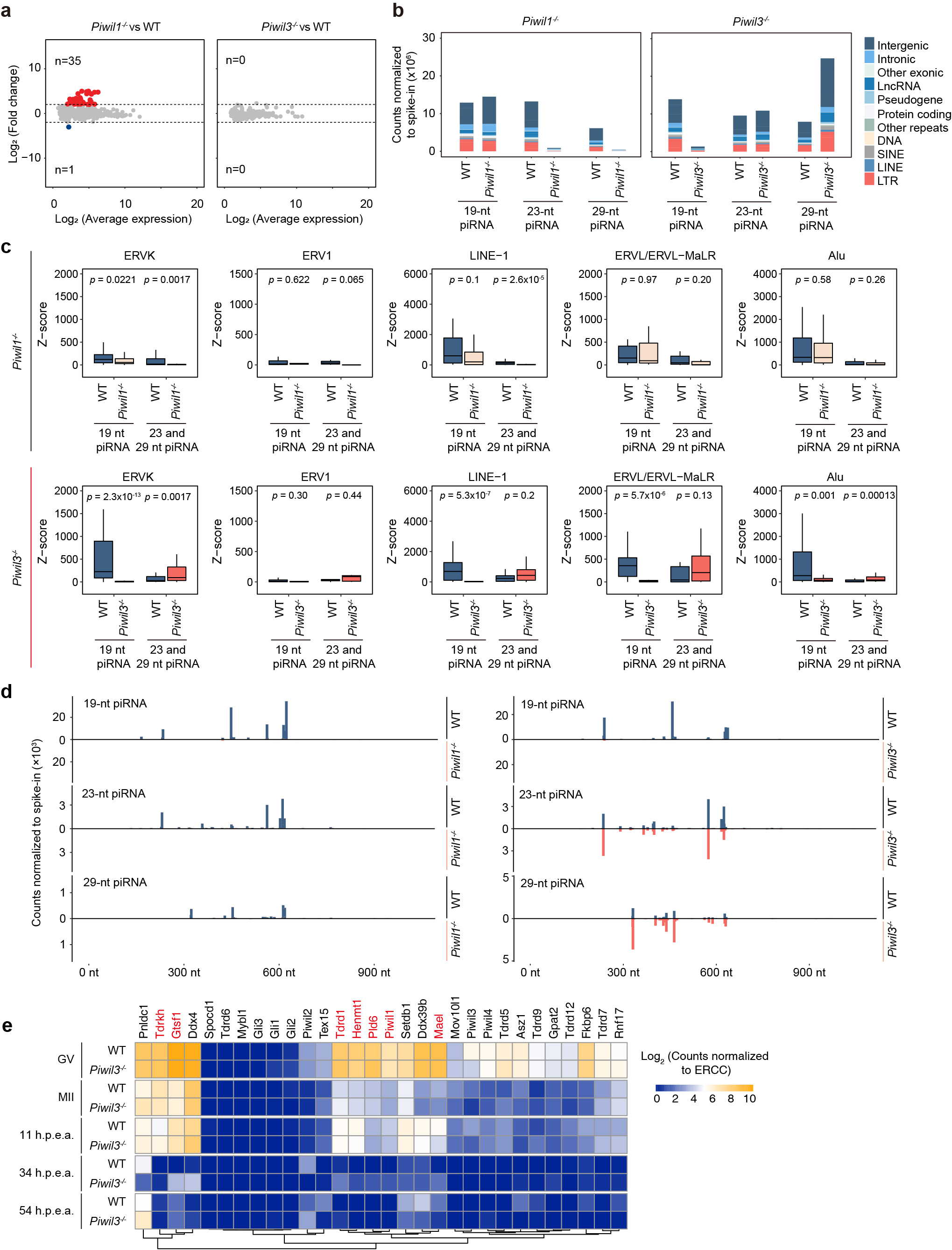


**Supplementary Fig. 5 Interplay between PIWIL1-piRNAs and PIWIL3-piRNAs on silencing TEs**

**(a)** Analysis of differentially expressed TEs in WT versus *Piwil1*^-/-^ or *Piwil3*^-/-^ MII oocytes. The expression levels of TEs were normalized to ERCC. The significantly up-regulated or down-regulated TE (≥ four-fold; FDR < 0.01, permutation test) are indicated in red or blue, respectively, and the TE number is shown. Data are the average values from three WT and *Piwil1*^-/-^ biological replicates or four WT and *Piwil3*^-/-^ biological replicates.

**(b)** Length and genomic annotation of piRNAs in WT versus *Piwil1*^-/-^ or *Piwil3*^-/-^ MII oocytes. piRNA counts were normalized to the exogenous spike-in.

**(c)** Box plots showing the Ping-Pong signature of 18-20-nt and 22-30-nt piRNAs derived from potentially active ERVK, L1, ERV1, Alu, or ERVL in WT or mutant MII oocytes. The level of the ping-pong signature is represented by the Z-score of 10-nt overlapped piRNAs from opposite strands; piRNAs with overlaps of different lengths serve as background. Z > 1.96 corresponds to P < 0.05. For the box plots, the centre line represents the median value, the box borders represent the upper and lower quartiles (25th and 75th percentiles, respectively), and the ends of the top and bottom whiskers represent the maximum and minimum scores, respectively. Two-sided Wilcoxon test was employed to examine the statistical significance, and no adjustments were made for multiple comparisons. Data are the average values of the biological replicates at each time point.

**(d)** Example (ltr-1_family-48|LTR/ERVK) of 19-nt, 23-nt, and 29-nt piRNAs distribution derived from ERVK families in WT, *Piwil1*^-/-^ and *Piwil3*^-/-^ MII oocytes. The horizontal axis represents the position of the consensus TE, and the vertical axis represents the normalized number of all mapped piRNAs with the same 5' end at each position.

**(e)** Heatmaps illustrating the expression of piRNA pathway genes during oogenesis and early embryogenesis in WT or *Piwil3*^-/-^ golden hamsters.

Data in (**b-e**) are the average values from three WT and two *Piwil1*^-/-^ biological replicates or four WT and *Piwil3*^-/-^ biological replicates.


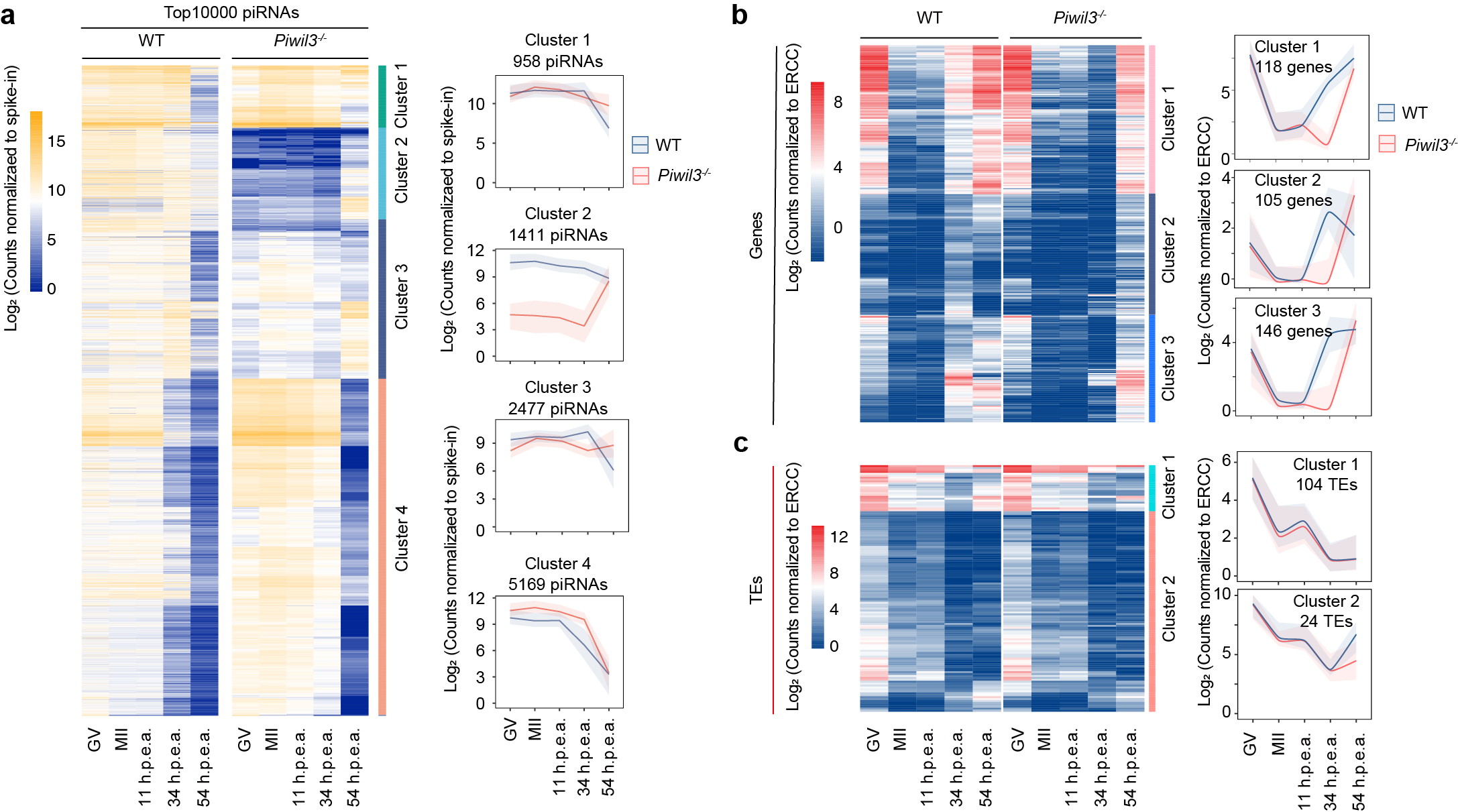


**Supplementary Fig. 6 Gene expression change in maternal *Piwil3****^-/-^* **embryos is delayed**

**(a-c)** Heatmaps showing the expression of the top 10000 piRNAs (**a**) or genes (**b**) or TEs **(c)** during oogenesis and early embryogenesis in WT or *Piwil3*^-/-^ golden hamsters. The displayed genes or TEs are up-regulated in WT 34 h.p.e.a. embryo versus WT 11 h.p.e.a. embryo. The clustering method ward is used. The line chart on the right illustrates the median value of piRNA expression on each selected cluster, the shadow borders represent the upper and lower quartiles (25th and 75th percentiles, respectively). The color of the bar on the right side of the heatmap represents different clusters.

Data in (**a-c**) are the average values of the biological replicates at each time point: n = 3 (GV), n = 4 (MII), n =5 (11 h.p.e.a.), n = 4 (34 h.p.e.a.), or n = 2 (54 h.p.e.a.) for WT; n =3 (GV), n = 4 (MII), n = 5 (11 h.p.e.a.), n = 3 (34 h.p.e.a.), or n = 2 (54 h.p.e.a.) for *Piwil3* mutants. *Piwil3*^-/-^, *Piwil3^-/-^* oocytes or maternal *Piwil3^-/-^* embryos.


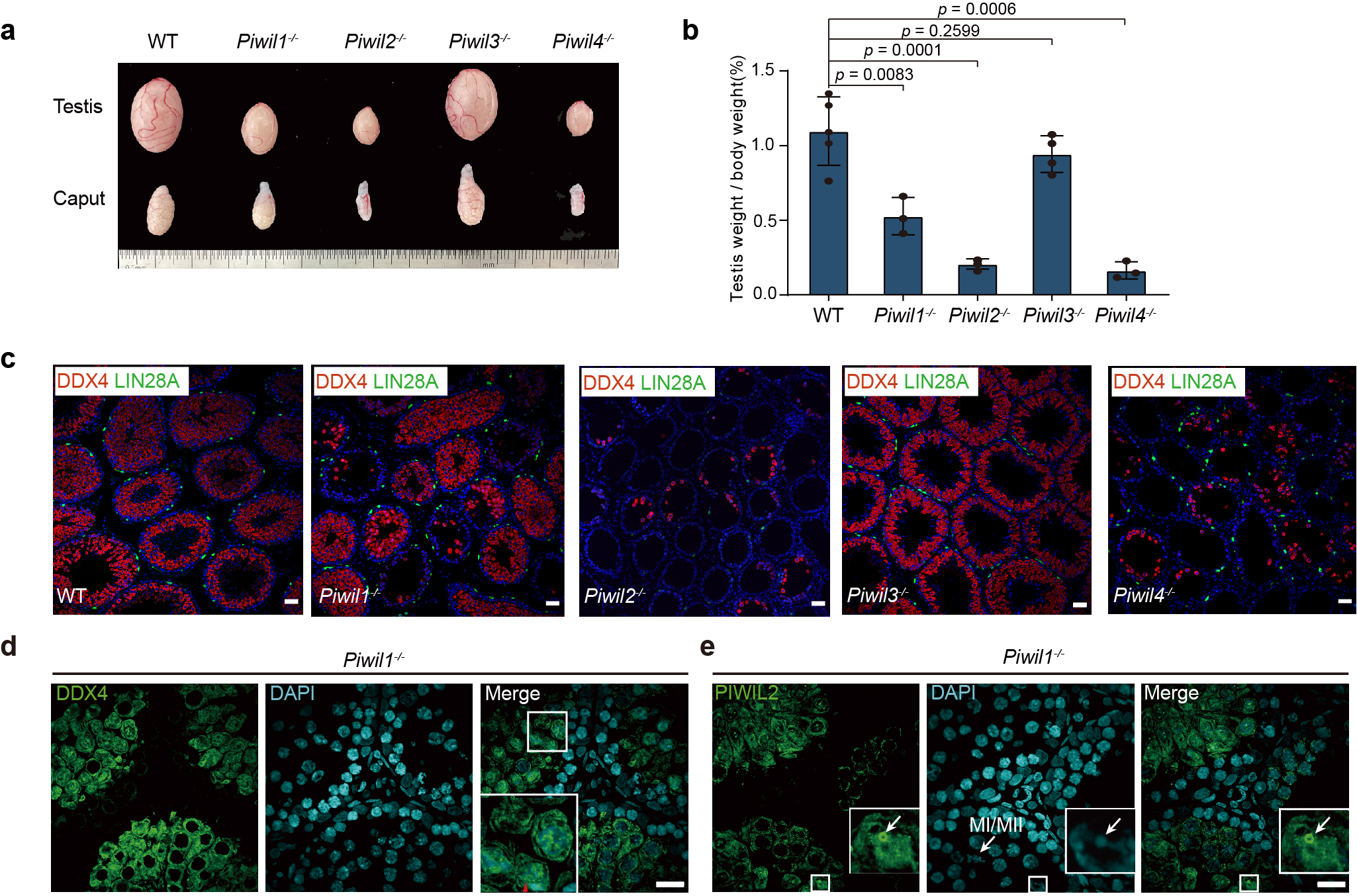


**Supplementary Fig. 7 Spermatogenesis defects in *Piwi*-deficient golden hamsters**

**(a)** Comparison of the testes and caput from adult WT, *Piwil1*^-/-^, *Piwil2*^-/-^, *Piwil3*^-/-^, and *Piwil4*^-/-^ golden hamsters.

**(b)** The weight ratio of the testis to body in WT or *Piwi*-deficient adult testes. Data are mean ± s.e.m. All *p* values are from two-tailed Student’s *t*-tests. n = 5 (WT), 3 (*Piwil1*^-/-^), 4 (*Piwil2*^-/-^), 4 (*Piwil3*^-/-^), or 3 (*Piwil4*^-/-^) biologically independent samples.

**(c)**  Immunofluorescence staining of adult testes to examine the presence of germ cells (marked by DDX4), and undifferentiated spermatogonia (marked by LIN28A). Diminished LIN28A staining was observed in *Piwil2*^-/-^ testes. Scale bars, 25 μm.

**(d-e)** Immunofluorescence staining of adult *Piwil1*^-/-^ testes with anti-DDX4 antibodies (**d**) and anti-PIWIL2 antibodies (**e**) shows the abnormal nuclear localization of DDX4 and PIWIL2. White arrows indicate aberrant perinuclear granules that resemble chromatoid bodies (CB). MI/MII, meiosis I/ meiosis II. Scale bars, 20 μm.

Source data are provided as a Source Data file.


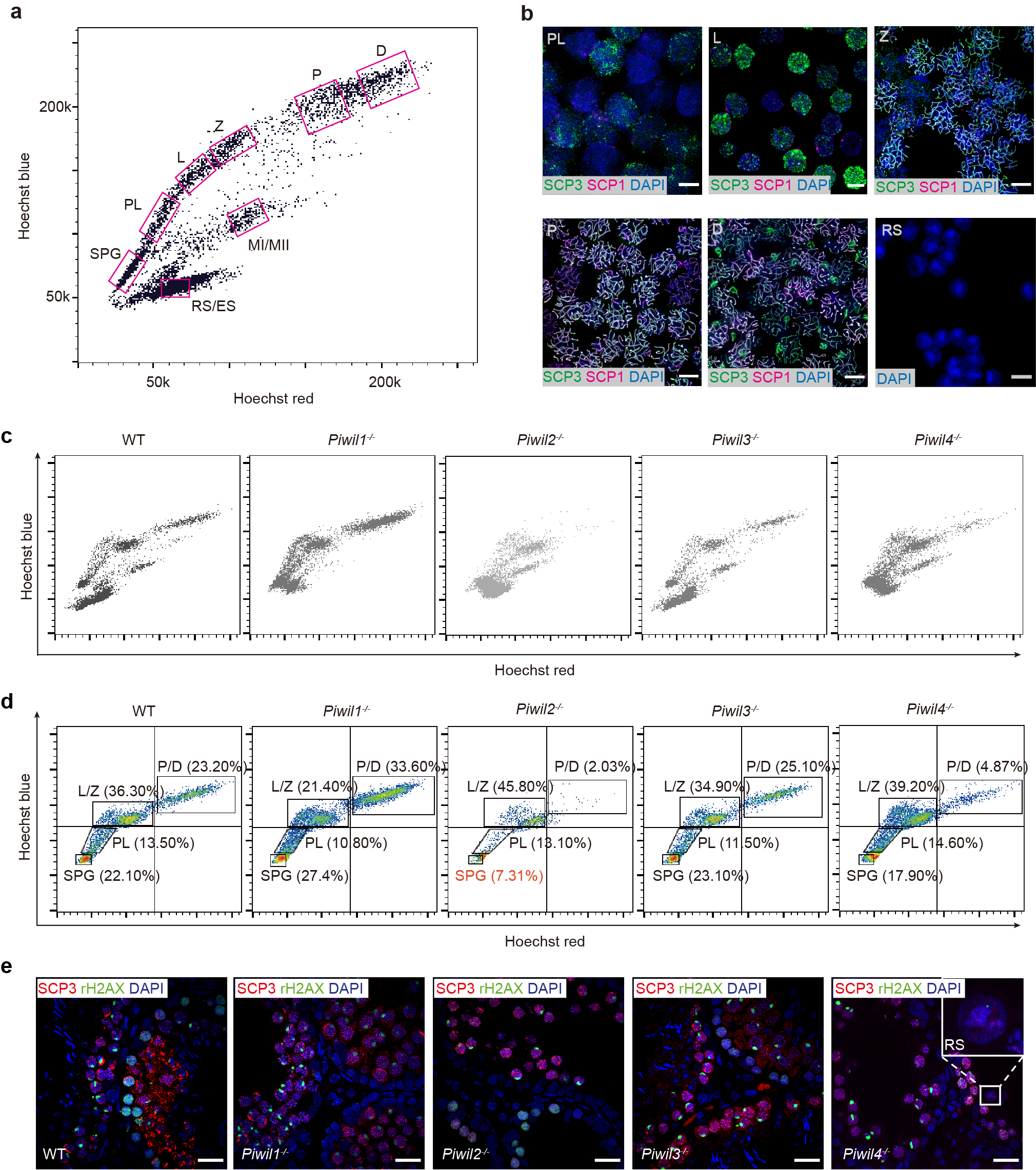


**Supplementary Fig. 8 Composition of spermatogenic cells in *Piwi*-deficient testes**

**(a)** FACS sorting of spermatocytes and round spermatids from the adult testes. Spermatogenic cells are stained with Hoechst33342, and main cell populations are indicated using the red boxes. SPG, spermatogonia; PL, preleptotene spermatocytes; L, leptotene spermatocytes; Z, zygotene spermatocytes; P, pachytene spermatocytes; D, diplotene spermatocytes; MI/MII, meiosis I/ meiosis II spermatocytes; RS/ES, round/elongating spermatids.

**(b)** Chromatin spread and immunofluorescence staining from purified spermatocytes and round spermatids in **(A)** to determine the cell purity. Spread nuclei are double-labeled with SCP1 and SCP3 and counterstained with DAPI. Scale bar, 8 µm.

**(c)** Flow cytometric analysis of adult hamster testicular cells. DNA was stained with Hoechst33342.

**(d)** Spermatocytes purified from the testicular cells in (**C**) and reanalyzed by FACS. The ratio of individual substages is indicated.

**(e)** Immunofluorescence analysis of adult hamster testes with anti-SCP3 and anti-γH2AX antibodies. Nuclei were stained with DAPI. Scale bars, 20 μm.


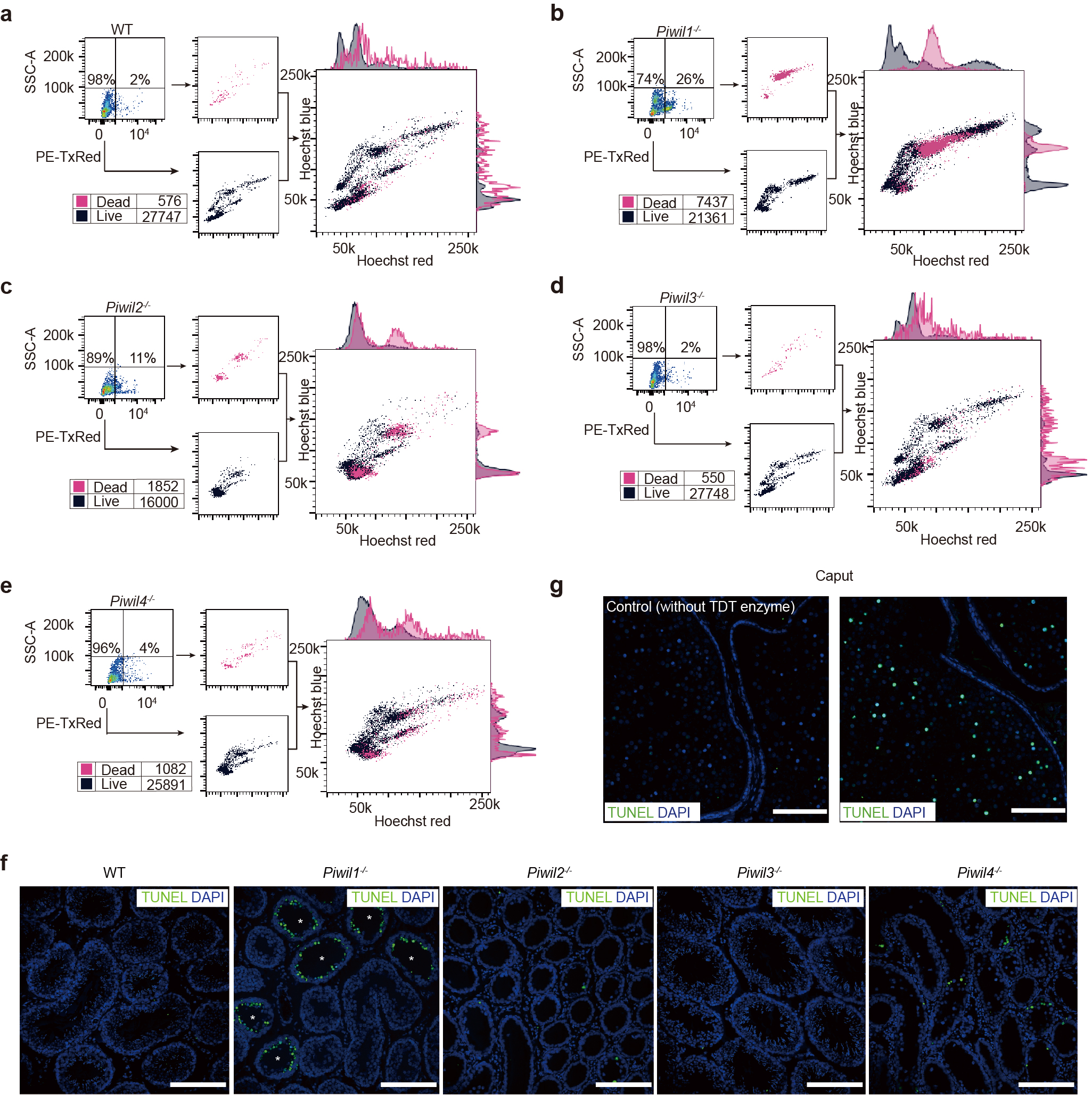


**Supplementary Fig. 9 Spermatocytes undergo mass mortality in *Piwil1****^-/-^* **males**

**(a-e)** Flow cytometric analysis of adult testicular cells from WT (**a**), *Piwil1*^-/-^ (**b**), *Piwil2*^-/-^ (**c**), *Piwil3*^-/-^ (**d**), and *Piwil4*^-/-^ (**e**) golden hamsters using Hoechst33342 and PI. Cell viability was determined by the fluorescence of “PE-TxRed”. Cells that fall within the bottom left gate are living (black), while within the bottom right are dead (magenta). The frequency of each event is present in the table. Those cells were further subdivided into different species based on the fluorescence of Hoechst.

**(f-g)** TUNEL staining analysis of adult testes **(f)** from WT, *Piwil1*^-/-^, *Piwil2*^-/-^, *Piwil3*^-/-,^ and *Piwil4*^-/-^ golden hamsters or caput epididymis **(g)** from *Piwil1*^-/-^ golden hamsters. Elevated levels of apoptosis in *Piwil1*^-/-^, *Piwil2*^-/-^, and *Piwil4*^-/-^ adult testis and *Piwil1*^-/-^ caput epididymis is observed. Scale bars, 200 μm.


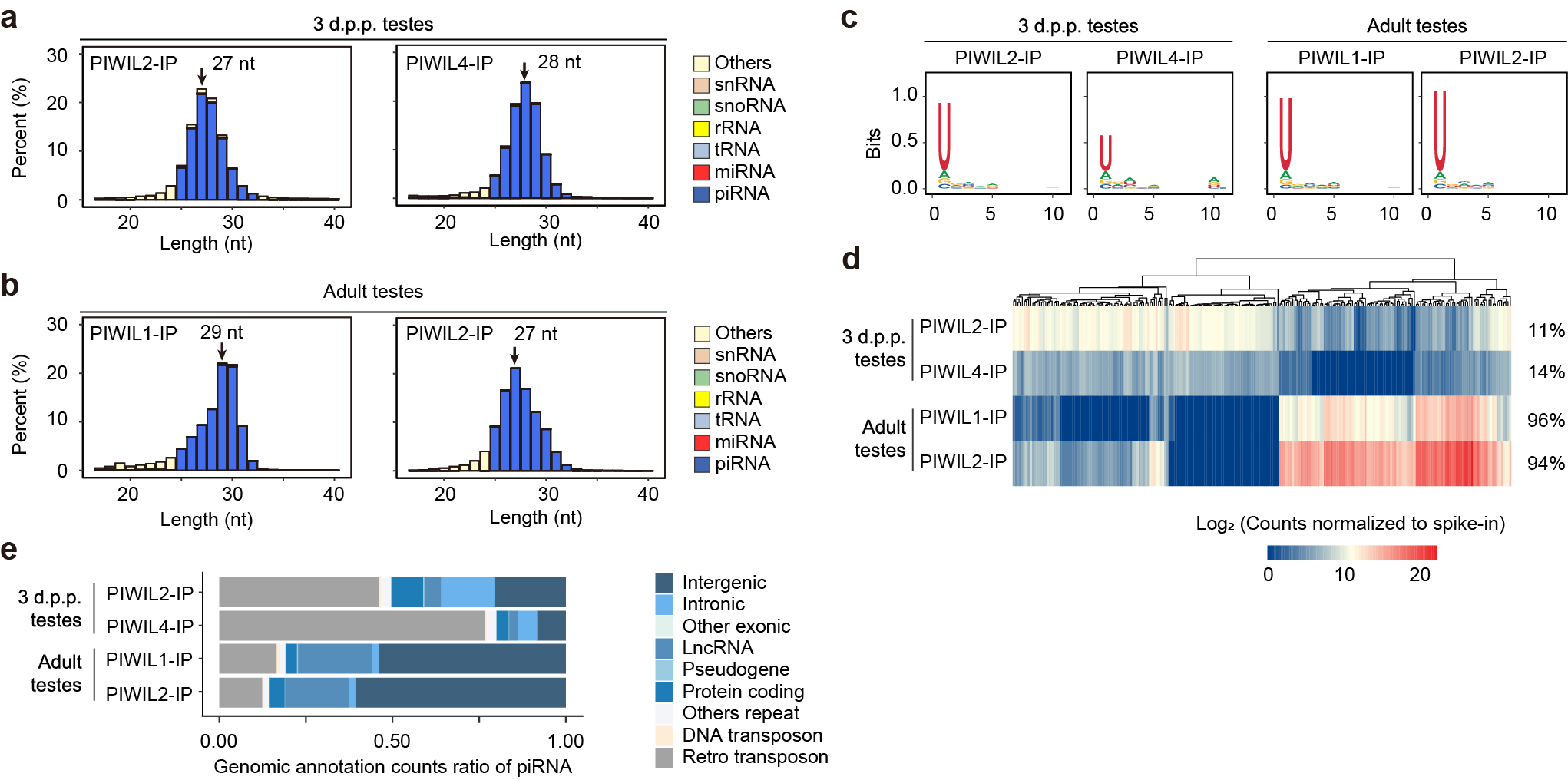


**Supplementary Fig. 10 Characterization of piRNAs in WT postnatal and adult testes**

**(a-b)** The size distribution of PIWI-bound piRNAs identified by immunoprecipitation using anti-PIWIL1, anti-PIWIL2, or anti-PIWIL4 antibodies in postnatal **(a)** or adult testes **(b)**. The small RNA counts were normalized to the exogenous spike-in. Data are the average values of two biological replicates.

**(c)** Base-preference of PIWIL1-, PIWIL2- and PIWIL4-piRNAs in postnatal or adult testes.

**(d)** The expression level of the top 100 piRNA clusters in postnatal or adult testes from immunoprecipitation. The normalization method is the same as **(a)**. The values on the right side of the heatmap indicate the ratio of piRNAs derived from these top 100 clusters to total piRNAs.

**(e)** Genomic annotation of PIWIL2- and PIWIL4-piRNAs in postnatal testes or PIWIL1- and PIWIL2-piRNAs in adult testes. piRNA origins are indicated by different colors.


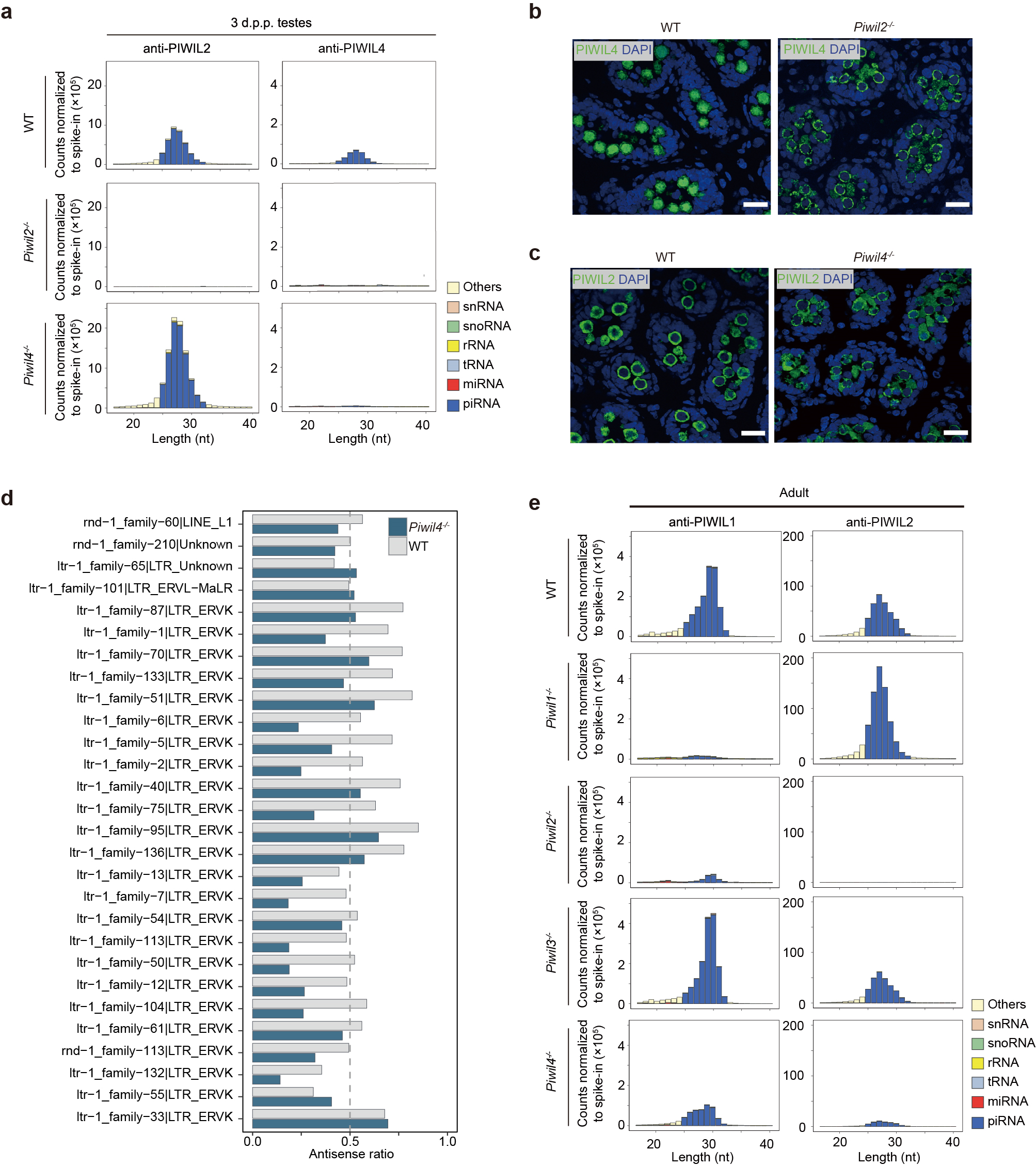


**Supplementary Fig. 11 Characterization of piRNAs in *Piwi*-deficient testes**

**(a)** The size distribution of PIWI-bound piRNAs identified by immunoprecipitation using anti-PIWIL2 or anti-PIWIL4 antibodies in WT or *Piwi*-deficient postnatal testes. Small RNA counts were normalized to the exogenous spike-in. Data are the average values of two biological replicates.

**(b-c)** Immunofluorescence analysis of *Piwi*-deficient postnatal testes with anti-PIWIL4 antibodies (**b**) or anti-PIWIL2 antibodies (**c**). PIWIL2 deficiency disturbs the nuclear localization of PIWIL4, while PIWIL4 deficiency does not change the expression or localization of PIWIL2. Scale bars, 20 μm.

**(d)** Proportion of piRNAs derived from TE antisense strands in WT or *Piwil4*^-/-^ postnatal testes. All up-regulated TEs in *Piwil2^-/-^* or *Piwil4^-/-^* postnatal testes are included. Data are the average values of two biological replicates.

(**e)** The size distribution of PIWI-bound piRNAs identified by immunoprecipitation using anti-PIWIL1 or anti-PIWIL2 antibodies in WT or *Piwi*-deficient adult testes. The normalization method and the number of biological replicates are the same as in **(a)**.


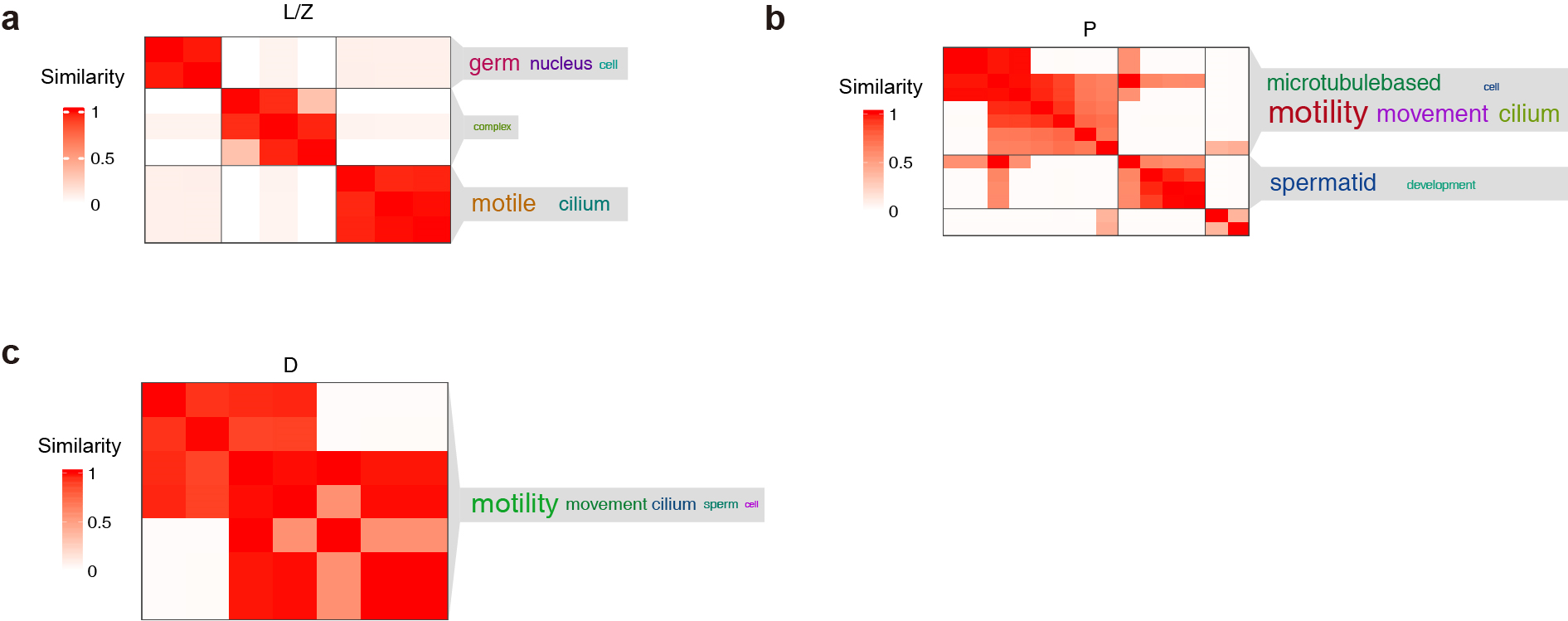


**Supplementary Fig. 12 Gene ontology (GO) analysis of DEGs in *Piwil1*^-/-^ spermatocytes**

**(a-c)** Top-ranking GO terms (biological processes) of differentially expressed genes in *Piwil1*-deficient leptotene/zygotene **(a)**, pachytene **(b)**, or diplotene **(c)** spermatocytes.


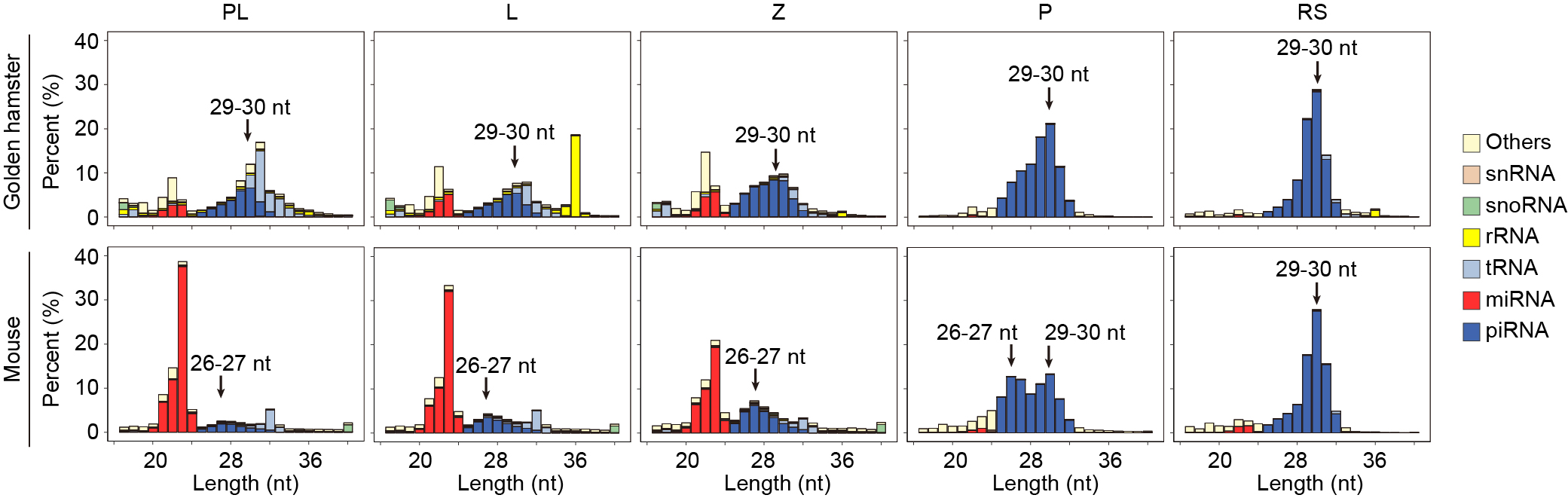


**Supplementary Fig. 13 Composition of small RNA categories during spermatogenesis in golden hamsters and mice**

The golden hamster germ cells of each stage were collected by FACS sorting. The small RNA counts were normalized to the total mapped reads. For golden hamsters, data are the average values of two biological replicates at each time point. For mice, data are obtained from GSE101933.
