# Supplementary material for: The non-redundant functions of PIWI family proteins in gametogenesis in golden hamsters
Source: Nat Commun. 2023 Aug 29;14:5267. doi: 10.1038/s41467-023-40650-x (PMC10465502; doi:10.1038/s41467-023-40650-x)
Supplement: Supplementary file 3 — Description of Additional Supplementary Files [file 41467_2023_40650_MOESM3_ESM.pdf]

Description of Additional Supplementary Files

Supplementary Data 1: List of oligonucleotide sequences.

Supplementary Data 2: Sequencing data summary of small- and long-RNAs

Supplementary Data 3: Gene expression levels in oocytes and early embryos derived from WT, *Piwil2*$^{-/-}$, *Piwil3*$^{-/-}$ and *Piwil4*$^{-/-}$ golden hamsters.

Supplementary Data 4: Gene expression levels in *Piwi*-deficient golden hamster testis.

Supplementary Data 5: Differentially expressed consensus TEs in WT versus *Piwil1*$^{-/-}$ MII oocyte.

Supplementary Data 6: Differentially expressed consensus TEs in WT versus *Piwil3*$^{-/-}$ MII oocyte.

Supplementary Data 7: Gene expression levels in oocytes and early embryos derived from WT and *Piwil3*$^{-/-}$ golden hamsters.

Supplementary Data 8: Top10,000 piRNA expression during oocyte and embryo development in WT or *Piwil3*$^{-/-}$ mutants.

Supplementary Data 9: List of delayed genes in maternal *Piwil3*$^{-/-}$ embryos

Supplementary Data 10: Consensus-TE expression levels in oocytes and early embryos derived from WT and *Piwil3*$^{-/-}$ golden hamsters.

Supplementary Data 11: Consensus-TE expression levels in *Piwi*-deficient golden hamster testis.

Supplementary Data 12: Gene expression levels in Piwil1-deficient spermatogenesis of golden hamster.

Supplementary Data 13: Consensus-TE expression levels in Piwil1-deficient spermatogenesis of golden hamster.

Supplementary Data 14: The pachytene piRNA clusters in spermatogenesis of golden hamster.

Supplementary Data 15: The syntenic genomic coordinates of the pachytene piRNA clusters in spermatogenesis of golden hamster for mouse and human.
