# Supplementary material for: The non-redundant functions of PIWI family proteins in gametogenesis in golden hamsters
Source: Nat Commun. 2023 Aug 29;14:5267. doi: 10.1038/s41467-023-40650-x (PMC10465502; doi:10.1038/s41467-023-40650-x)
Supplement: Supplementary file 19 — Reporting Summary [file 41467_2023_40650_MOESM19_ESM.pdf]

nature portfolio

Corresponding author(s): Aimin Shi, Jianmin Li, Ligang Wu

Last updated by author(s): Jul 24, 2023

Reporting Summary

Nature Portfolio wishes to improve the reproducibility of the work that we publish. This form provides structure for consistency and transparency in reporting. For further information on Nature Portfolio policies, see our Editorial Policies and the Editorial Policy Checklist.

Please do not complete any field with "not applicable" or n/a. Refer to the help text for what text to use if an item is not relevant to your study.
For final submission: please carefully check your responses for accuracy; you will not be able to make changes later.

Statistics

For all statistical analyses, confirm that the following items are present in the figure legend, table legend, main text, or Methods section.

n/a	Confirmed	
☐	☒	The exact sample size (*n*) for each experimental group/condition, given as a discrete number and unit of measurement
☐	☒	A statement on whether measurements were taken from distinct samples or whether the same sample was measured repeatedly
☐	☒	The statistical test(s) used AND whether they are one- or two-sided<br>*Only common tests should be described solely by name; describe more complex techniques in the Methods section.*
☒	☐	A description of all covariates tested
☐	☒	A description of any assumptions or corrections, such as tests of normality and adjustment for multiple comparisons
☐	☒	A full description of the statistical parameters including central tendency (e.g. means) or other basic estimates (e.g. regression coefficient) AND variation (e.g. standard deviation) or associated estimates of uncertainty (e.g. confidence intervals)
☐	☒	For null hypothesis testing, the test statistic (e.g. *F*, *t*, *r*) with confidence intervals, effect sizes, degrees of freedom and *P* value noted<br>*Give P values as exact values whenever suitable.*
☒	☐	For Bayesian analysis, information on the choice of priors and Markov chain Monte Carlo settings
☒	☐	For hierarchical and complex designs, identification of the appropriate level for tests and full reporting of outcomes
☒	☐	Estimates of effect sizes (e.g. Cohen's *d*, Pearson's *r*), indicating how they were calculated

Our web collection on statistics for biologists contains articles on many of the points above.

Software and code

Policy information about availability of computer code

Data collection	Sequencing data were collected on illumina HiSeq X10 or Nova-seq.
Data analysis	For smallRNA-seq:Raw read1 fastq file was used to identify small RNAs, which were preprocessed using the FASTX Toolkit 0.0.14. The preprocessing steps are as follows: intercept the first 50bp of reads and filter out low-quality sequences (-q 20, -p 90); After quality-filtering, sequencing reads were clipped from the 3`adapter allowing for a minimum match of 10nt from the 5` ends. Reads unable to match the adapter sequence or with lengths shorter than 17 bp were discarded, and the remaining reads were mapped to genome by bowtie ((hamster: BCM_Maur_2.0, mouse: mm10), bowtie version 1.2.1.1, parameters -k=100 -v=0). If the reads mapped more than 100 loci to the genome, 100 of all mapped loci are randomly output. The reads were aligned sequentially to known miRNA, tRNA, rRNA, snoRNA, and snRNA sequences by Bowtie without any mismatch allowed (bowtie parameter is -k=100 -v=0 -norc) and reads that aligned to pre-miRNA were identified as miRNAs. The remaining reads of the specified length were extracted and used to identify piRNAs according to the previously described method 46 with slight modifications. The length of candidate piRNAs in male samples is 25~32nt, and the length of candidate piRNAs in female samples is 17~32nt. The clustering parameters were determined as MinReads = 5 and Eps = 2500 bp by running a series of k-dist analyses of our data with different Eps and MinReads. All candidate clusters that satisfied these parameters were considered piRNA clusters, and the remaining sequences located in these clusters were defined as piRNAs without any further filtering. Reads in each library were normalized by sequencing depth or read counts of miRNAs or read counts of exogenous spike-in.<br>For RNA-seq:The raw paired-end fastq reads had adapters removed and were quality-filtered by TrimGalore-0.5.0 with the parameters --paired --quality 20 --phred33 --stringency 1 --length 35. The remaining reads were mapped to the genome or identified TE by STAR v2.9. Uniquely mapped reads and multiple mapped reads with fewer than 10,000 genome copies were used for downstream analysis. The specific alignment parameters are as follows: --winAnchorMultimapNmax 10000 --outFilterMultimapNmax 10000 --alignSJoverhangMin 8 --alignSJDBoverhangMin 1 --outFilterMismatchNmax 999 --alignIntronMin 20 --alignIntronMax 1000000 --alignMatesGapMax 1000000 --twopassMode Basic --outSAMprimaryFlag AllBestScore. Calculation of the read count of known genes or TEs was performed by featureCounts

v1.6.4 with the parameters -p -B -C -M -Q 20. The expression of known genes or TEs was normalized to the ERCC or mapped reads. Genes or TEs with normalized count>2 in 40% or more of samples were used for differential analysis. Differentially expressed genes or TEs were identified using the permutation test and the derived p values were adjusted for multiple testing using the Benjamini-Hochberg procedure (≥ 4-fold change and FDR <0.01).

For manuscripts utilizing custom algorithms or software that are central to the research but not yet described in published literature, software must be made available to editors and reviewers. We strongly encourage code deposition in a community repository (e.g. GitHub). See the Nature Portfolio guidelines for submitting code & software for further information.

Data

Policy information about availability of data

All manuscripts must include a data availability statement. This statement should provide the following information, where applicable:

- Accession codes, unique identifiers, or web links for publicly available datasets
- A description of any restrictions on data availability
- For clinical datasets or third party data, please ensure that the statement adheres to our policy

All deep-sequencing data have been deposited at the National Center for Biotechnology Information NCBI Gene Expression Omnibus (GEO) (http://www.ncbi.nlm.nih.gov/geo/) database under accession number GSE217621 (https://www.ncbi.nlm.nih.gov/geo/query/acc.cgi?acc=GSE217621). The following publicly available datasets were used: human adult testis data GSE135791 (https://www.ncbi.nlm.nih.gov/geo/query/acc.cgi?acc=GSE135791); mouse adult testis data PRJNA421205 (https://www.ncbi.nlm.nih.gov/bioproject/PRJNA421205); mouse spermatogenesis data GSE101933 (https://www.ncbi.nlm.nih.gov/geo/query/acc.cgi?acc=GSE101933); Piwil1-/- related data GSE169528 (https://www.ncbi.nlm.nih.gov/geo/query/acc.cgi?acc=GSE169528). Source Data are provided with this paper.

Research involving human participants, their data, or biological material

Policy information about studies with human participants or human data. See also policy information about sex, gender (identity/presentation), and sexual orientation and race, ethnicity and racism.

Reporting on sex and gender	N/A
Reporting on race, ethnicity, or other socially relevant groupings	N/A
Population characteristics	N/A
Recruitment	N/A
Ethics oversight	N/A

Note that full information on the approval of the study protocol must also be provided in the manuscript.

Field-specific reporting

Please select the one below that is the best fit for your research. If you are not sure, read the appropriate sections before making your selection.

☒ Life sciences ☐ Behavioural & social sciences ☐ Ecological, evolutionary & environmental sciences

Life sciences study design

All studies must disclose on these points even when the disclosure is negative.

Sample size

No statistical method was used to predetermine sample size. The sample size was chosen based on previous experience and similar studies to reliably measure experimental parameter according to standards in the relevant field.
Relevant reference:
1.Yang, Q. et al. Single-cell CAS-seq reveals a class of short PIWI-interacting RNAs in human oocytes. Nature communications 10, 3389 (2019).
2.Carmell, M.A. et al. MIWI2 is essential for spermatogenesis and repression of transposons in the mouse male germline. Developmental cell 12, 503-514 (2007).
3.Kuramochi-Miyagawa, S. et al. Mili, a mammalian member of piwi family gene, is essential for spermatogenesis. Development 131, 839-849 (2004).
4.Deng, W. & Lin, H.F. miwi, a murine homolog of piwi, encodes a cytoplasmic protein essential for spermatogenesis. Developmental cell 2, 819-830 (2002).
5.Watanabe, T. et al. MITOPLD Is a Mitochondrial Protein Essential for Nuage Formation and piRNA Biogenesis in the Mouse Germline. Developmental cell 20, 364-375 (2011).
6.Huang, H.Y. et al. piRNA-Associated Germline Nuage Formation and Spermatogenesis Require MitoPLD Profusogenic Mitochondrial-Surface Lipid Signaling. Developmental cell 20, 376-387 (2011).
7.Zheng, K. et al. Mouse MOV10L1 associates with Piwi proteins and is an essential component of the Piwi-interacting RNA (piRNA)

pathway. Proceedings of the National Academy of Sciences of the United States of America 107, 11841-11846 (2010).
8.Frost, R.J.A. et al. MOV10L1 is necessary for protection of spermatocytes against retrotransposons by Piwi-interacting RNAs. Proceedings of the National Academy of Sciences of the United States of America 107, 11847-11852 (2010).
The followings are short descriptions of sample size in our experiments.
1. The matting performance was performed by using:
1) 10 WT males and 10 WT females that give birth to 21 litters;
2) 8 PIWIL1(-/-) males and 8 WT females that give birth to 0 litters;
3) 6 PIWIL2(-/-) males and 6 WT females that give birth to 0 litters;
4) 8 PIWIL3(-/-) males and 8 PIWIL3(+/-) females that give birth to 14 litters;
5) 6 PIWIL4(-/-) males and 6 WT females that give birth to 0 litters;
6) 8 WT males and 8 PIWIL1(-/-) females that give birth to 0 litter;
7) 5 WT males and 5 PIWIL2(-/-) females that give birth to 5 litter;
8) 5 PIWIL3(+/-) males and 5 PIWIL3(-/-) females that give birth to 6 litter;
9) 9 PIWIL3(-/-) males and 9 PIWIL3(-/-) females that give birth to 8 litter;
10) 5 WT males and 5 PIWIL4(-/-) females that give birth to 5 litter;
2. The statistics of embryo stages in vivo and in vitro were performed by using:
1) 36 WT embryos and 27 PIWIL3(-/-) embryos in vivo;
2) 15 WT embryos and 28 PIWIL3(-/-) embryos in vitro;
3. The statistics of abortion rate were performed by using 16 PIWIL3(+/-) and 9 PIWIL1(-/-) golden hamster.
4. The number of super-ovulated oocytes was performed by using 10 WT,10 PIWIL1(-/-),4 PIWIL2(-/-) ,10 PIWIL3(-/-)and 4 PIWIL4(-/-) golden hamsters.
5. The small RNA and mRNA sequencing of oocytes and embryos at 8 developmental stages were performed by using 2-6 replicates. The detail information was list in the supplementary table 2.

Data exclusions: No data were excluded from the analysis.

Replication: All experiments were independently repeated at least twice with similar results. And the number of biological replicates and the number of animals are indicated in Figure legends and methods section.

Randomization: All samples were allocated randomly, including the collection of oocytes and embryos, library construction, sample pooling, and data collection.

Blinding: The investigators were blinded to group allocation during constructing sequencing libraries. No other blinding was involved, but randomization was used. Otherwise, all data analyses were performed by unbiased software programs.

Reporting for specific materials, systems and methods

We require information from authors about some types of materials, experimental systems and methods used in many studies. Here, indicate whether each material, system or method listed is relevant to your study. If you are not sure if a list item applies to your research, read the appropriate section before selecting a response.

Materials & experimental systems

n/a	Involved in the study	
☐	☒	Antibodies
☐	☒	Eukaryotic cell lines
☒	☐	Palaeontology and archaeology
☐	☒	Animals and other organisms
☒	☐	Clinical data
☒	☐	Dual use research of concern
☒	☐	Plants

Methods

n/a	Involved in the study	
☒	☐	ChIP-seq
☐	☒	Flow cytometry
☒	☐	MRI-based neuroimaging

Antibodies

Antibodies used

Antibody	Clonality	Manufacturer	Catalogue number (clone)	Dilutions	Used
Anti-Alpha Tubulin	Mouse monoclonal	Proteintech	66031-1-lg (1E4C11)	1:200	Wes-Simple
Anti-β-actin	Mouse monoclonal	Proteintech	66009-1-lg (2D4H5)	1:200	Wes-Simple
Anti-PIWIL1	Rabbit polyclonal	Homemade	See methods	1:100	Wes-Simple
Anti-PIWIL2	Mouse monoclonal	Millipore	MABE363 (13E-3)	1:100	Wes-Simple
Anti-PIWIL3	Rabbit polyclonal	Homemade	See methods	1:100	Wes-Simple
Anti-PIWIL4	Rabbit polyclonal	Homemade	See methods	1:100	Wes-Simple
Anti-PIWIL1	Rabbit polyclonal	Homemade	See methods	1:200	IF
Anti-PIWIL2	Mouse monoclonal	Millipore	MABE363 (13E-3)	1:200	IF
Anti-PIWIL3	Rabbit polyclonal	Homemade	See methods	1:200	IF
Anti-PIWIL4	Rabbit polyclonal	Homemade	See methods	1:200	IF
Anti-DCP1A	Mouse monoclonal	SANTA CRUZ	Cat#sc-100706 (56-Y)	1:200	IF
Anti-TDRKH	Sheep Polyclonal	Thermo Fisher	PA5-48098	1:80	IF
Anti-DDX6	Mouse monoclonal	SANTA CRUZ	sc-376433 (E-12)	1:100	IF
Anti-TDRD1	Rat monoclonal	R&D	MAB6296 (739206)	1:50	IF
Anti-ATP5A1	Mouse monoclonal	Proteintech	66037-lg (1B10H3)	1:200	IF

Anti-LIN28A	Rabbit monoclonal	CST	8641 (D1A1A)	1:200	IF
Anti-DDX4	Mouse monoclonal	Abcam	ab27591	1:200	IF
Anti-SCP3	Mouse monoclonal	SANTA CRUZ	sc-74569 (clone D-1)	1:500	IF
Anti-SCP3	mouse polyclonal	Abcam	ab15093	1:500	IF
Anti-SCP1	Rabbit polyclonal	Abcam	ab15090	1:500	IF
Anti-γH2AX	Mouse monoclonal	Millipore	05-636 (JBW301)	1:1,000	IF
Anti-mouse IgG-Alexa488	Donkey polyclonal	Thermo Fisher	A-21202	1:800	IF
Anti-rabbit IgG-Alexa488	Goat polyclonal	Thermo Fisher	A-11034	1:500	IF
Anti-rabbit IgG-CY3	Donkey polyclonal	Jackson IR	711-165-152	1:400	IF
Anti-rabbit IgG-CY3	Donkey polyclonal	Jackson IR	713-165-147	1:400	IF
Anti-tubulin FITC	Mouse monoclonal	Sigma	F2168 (DM1A)	1:500	IF
Anti-rabbit IgG-Alexa594	Donkey polyclonal	Thermo Fisher	A21207	1:200	IF
Anti-rabbit IgG-CF647	Donkey polyclonal	Sigma	A37573	1:1,000	IF
Anti-PIWIL1	Rabbit polyclonal	Homemade	See methods	1.5 μg	RIP
Anti-PIWIL2	Mouse monoclonal	Millipore	MABE363 (13E-3)	1μg	RIP
Anti-PIWIL3	Rabbit polyclonal	Homemade	See methods	1μg	RIP
Anti-PIWIL4	Rabbit polyclonal	Homemade	See methods	1μg	RIP
Anti-Flag-HRP	mouse monoclonal	Sigma	A8592 (M2)	1:5000	WB

Validation

All commercial antibody lots are routinely tested by the manufacturers and we validate their specificity in human cells and golden hamster. The references and manufacturer validations are list as follows:
1.Anti-β-actin: https://www.ptglab.com/products/Pan-Actin-Antibody-66009-1-Ig.htm#publications
2.Anti-PIWIL1: Yang, Q. et al. Single-cell CAS-seq reveals a class of short PIWI-interacting RNAs in human oocytes. Nature communications 10, 3389 (2019).
3.Anti-DCP1A: https://www.scbt.com/p/dcp1a-antibody-56-y?requestFrom=search
4.Anti-DDX6: https://www.scbt.com/p/rck-antibody-e-12?requestFrom=search
5.Anti-ATP5A1: https://www.ptgcn.com/products/ATP5A1-Antibody-66037-1-Ig.htm
6.Anti-TDRKH: https://www.thermofisher.cn/cn/zh/antibody/product/TDRKH-Antibody-Polyclonal/PA5-48098
7.Anti-SCP3: https://www.scbt.com/p/scp-3-antibody-d-1
8.Anti-SCP1: https://www.abcam.com/scp1-antibody-ab15090.html
9.Anti-γH2AX: https://www.merckmillipore.com/CN/zh/product/Anti-phospho-Histone-H2A.X-Ser139-Antibody-clone-JBW301,MM_NF-05-636?ReferrerURL=https%3A%2F%2Fwww.google.com.hk%2F&bd=1#documentation
10.Anti-PIWIL3: Yang, Q. et al. Single-cell CAS-seq reveals a class of short PIWI-interacting RNAs in human oocytes. Nature communications 10, 3389 (2019).
11.Anti-rabbit IgG-HRP conjugate: https://www.thermofisher.cn/cn/zh/antibody/product/Goat-anti-Rabbit-IgG-H-L-Secondary-Antibody-Polyclonal/31466
12.Anti-mouse IgG-HRP conjugate:https://www.thermofisher.cn/cn/zh/antibody/product/Goat-anti-Mouse-IgG-H-L-Secondary-Antibody-Polyclonal/31430
13. Anti-mouse IgG-Alexa488: https://www.thermofisher.cn/cn/zh/antibody/product/Donkey-anti-Mouse-IgG-H-L-Highly-Cross-Adsorbed-Secondary-Antibody-Polyclonal/A-21202
14.Anti-rabbit IgG-Alexa488: https://www.thermofisher.cn/cn/zh/antibody/product/Goat-anti-Rabbit-IgG-H-L-Highly-Cross-Adsorbed-Secondary-Antibody-Polyclonal/A-11034
15.Anti-rabbit IgG-CY3: https://www.jacksonimmuno.com/catalog/products/711-165-152
16.Anti-tubulin FITC: https://www.sigmaaldrich.cn/CN/en/product/sigma/f2168?context=product
17.Anti-rabbit IgG-Alexa594: https://www.thermofisher.cn/antibody/product/A-21207.html?
18.Anti-rabbit IgG-CF647: https://www.sigmaaldrich.cn/CN/zh/product/sigma/sab4600177

Eukaryotic cell lines

Policy information about cell lines and Sex and Gender in Research

Cell line source(s)

We used the HEK293T (CRL-3216, ATCC) for the verification of PIWI antibodies.

Authentication

No additional authentication was performed on the HEK293T cells. Of note, these cells were only used for verifying the specificity of PIWI antibodies and not for biological/cell analysis.

Mycoplasma contamination

Cell line is tested every 2 months and all cells were mycoplasma negative.

Commonly misidentified lines (See ICLAC register)

No misidentified lines were used in the study.

Animals and other research organisms

Policy information about studies involving animals; ARRIVE guidelines recommended for reporting animal research, and Sex and Gender in Research

Laboratory animals

4-8 weeks old male and female Golden (Syrian) hamsters were purchased from Vital River (Charles River, China) and Liaoning Changshen Biotechnology Co. Ltd, and maintained under 14-h/10-h or 12-h/12-h light/dark cycles .
6-10 weeks old female WT golden hamsters were used to collect oocytes and embryos for producing TRY, PIWIL1 , PIWIL2, PIWIL3 and PIWIL4 golden hamsters.
6-10 weeks old female TRY-deficient golden hamsters were used as surrogate hamsters to produce PIWIL1 , PIWIL2, PIWIL3 and PIWIL4 golden hamsters.
8-20 weeks old female WT and PIWIL1-4(-/-) were used to collect oocytes and embryos for single oocyte small RNA and mRNA library

constuction (Fig. 3,Fig.4A-B and Extended Data Fig. 3D,3E,S5,S6), Western blotting (Fig. 2A and Extended Data Fig. 4E,4F), immunostaining (Fig. 1C-F,2B and Extended Data Fig. 2A-C, 2F-H), transmission electron analysis (Extended Data Fig. 2D,E) and embryogenesis analysis (Fig. 4C,4D and Extended Data Fig. 4B).
8-20 weeks old female PIWIL1-4(-/-) were used in PAS staining (Extended Data Fig. 4A).
8-20 weeks old male WT and PIWIL1-4(-/-) were used in single oocyte small RNA and mRNA library constuction(Fig. 6,7 and Extended Data Fig.10,11A,11D,11E,12,13), PAS staining (Fig. 5A), FACS (Fig. 5C and Extended Data Fig.8A,8C,8D,9A-E), TUNEL staining (Extended Data Fig.9G and 9H) and immunostaining (Fig.1A,1B,2B and Extended Data Fig.1B-G,7C,7D,8B,8E,11B,11C) of testes, epitymus head and tail.
14, 21 and 40 days post partum (dpp) WT and PIWIL1,2,4(-/-) were used in PAS staining of testes Fig. 5B)

Wild animals	This study did not involve wild animals.
Reporting on sex	This study was conducted using both male and female golden hamsters.
Field-collected samples	This study did not involve field-collected samples.
Ethics oversight	All experiments were approved by the Nanjing Medical University Institutional Animal Care and Research Committee (IACUC code: 16120401).

Note that full information on the approval of the study protocol must also be provided in the manuscript.

Flow Cytometry

Plots

Confirm that:

☒ The axis labels state the marker and fluorochrome used (e.g. CD4-FITC).

☒ The axis scales are clearly visible. Include numbers along axes only for bottom left plot of group (a 'group' is an analysis of identical markers).

☒ All plots are contour plots with outliers or pseudocolor plots.

☒ A numerical value for number of cells or percentage (with statistics) is provided.

Methodology

Sample preparation	Testes were collected from mutant and WT control golden hamsters, and single cell suspensions were generated. Testicular cells were stained with Hoechst33342 and Propidium Iodide or not, washed and resuspended in DMEM medium containing 1%FBS
Instrument	BD FACSAria SORP(BD Biosciences)
Software	FlowJo(v10.4)
Cell population abundance	100 thousands single cells were analyzed from control and mutant testicular cells, respectively. 60 thousands live and single cells of each stage were obtained for sequencing from control and PIWIL1-mutant testicular cells.
Gating strategy	Testicular cells were stained with 1 ug ml-1 Propidium Iodide to exclude dead cells and stained with Hoechst33342 dye to quantify DNA content. The gating strategy is provided in the Fig. s8.

☒ Tick this box to confirm that a figure exemplifying the gating strategy is provided in the Supplementary Information.

This checklist template is licensed under a Creative Commons Attribution 4.0 International License, which permits use, sharing, adaptation, distribution and reproduction in any medium or format, as long as you give appropriate credit to the original author(s) and the source, provide a link to the Creative Commons license, and indicate if changes were made. The images or other third party material in this article are included in the article's Creative Commons license, unless indicated otherwise in a credit line to the material. If material is not included in the article's Creative Commons license and your intended use is not permitted by statutory regulation or exceeds the permitted use, you will need to obtain permission directly from the copyright holder. To view a copy of this license, visit http://creativecommons.org/licenses/by/4.0/
